# A Provincial Survey on the Perioperative Rehabilitation Needs and Experiences of Women Diagnosed with Breast Cancer

**DOI:** 10.3390/healthcare13243239

**Published:** 2025-12-10

**Authors:** Janny Mathieu, Charles Tétreau, Sandrine Jobin, Annabelle Doyon, Marie-Hélène Morin, Andrée-Anne Marchand, Martin Descarreaux

**Affiliations:** 1 Department of Anatomy, Université du Québec à Trois-Rivières, Trois-Rivières, QC G9A 5H7, Canada; 2 Department of Human Kinetics, Université du Québec à Trois-Rivières, 3351, Boul. des Forges, C.P. 500, Trois-Rivières, QC G8Z 4M3, Canada; charles.tetreau@uqtr.ca (C.T.);; 3 Psychosociology and Social Work Department, Université du Québec à Rimouski, Rimouski, QC G5L 3A1, Canadamarie-helene_morin@uqar.ca (M.-H.M.); 4 Department of General Surgery, Centre Intégré Universitaire de Santé et de Services Sociaux de la Mauricie-et-du-Centre-du-Québec, Trois-Rivières, QC G8Z 4M3, Canada; 5 Chiropractic Department, Université du Québec à Trois-Rivières, Trois-Rivières, QC G9A 5C5, Canada; andree-anne.marchand@uqtr.ca

**Keywords:** breast cancer, mastectomy, rehabilitation, access to care, social determinants of health

## Abstract

**Background/Objectives**: The breast cancer care continuum is associated with a physical and emotional burden resulting in significant needs for rehabilitation. A growing concern has been the underutilization of rehabilitation services, even when they are accessible, highlighting that several women’s needs remain unmet. The aim of this study was to investigate breast cancer patients’ physical and psychological rehabilitation needs and experiences, and to document the barriers encountered throughout the breast cancer care continuum. **Methods**: A cross-sectional study consisting of a self-reported online survey was conducted. Women aged 18 years and over with a diagnosis of breast cancer, who underwent breast surgery in the last 5 years in Quebec province, Canada, were eligible for this study. A questionnaire was developed by research team members, and content validity was evaluated by an expert group including professionals in rehabilitation, oncological care, and two patient partners. The questionnaire was completed once by each participant on the online platform Qualtrics ©. Closed-ended and open-ended questions were used to collect information on (1) sociodemographic characteristics, (2) clinical profiles, (3) rehabilitation needs, and (4) rehabilitation experiences. **Results**: The sample included participants from all 17 administrative regions of the Quebec province, 78.0% of whom lived in urban areas. Four hundred and four participants (78.4%) expressed rehabilitation needs, but only two hundred and forty-five participants (47.2%) reported the use of rehabilitation services. A lack of awareness of the service’s availability, the fact that the service was not prescribed or recommended, and financial constraints were cited as major barriers to accessing rehabilitation services. **Conclusions**: Breast cancer patients expressed significant needs for rehabilitation services but demonstrated low utilization rates, reflecting barriers to rehabilitation. Addressing the fragmented integration of rehabilitation services into the breast cancer care continuum and inequities in access based on social determinants of health should be prioritized.

## 1. Introduction

In 2024, breast cancer is projected to be the second most diagnosed cancer in Canada, while remaining the leading cancer diagnosis among women [1]. Efforts in screening, diagnosis and development of innovative targeted treatments reduced breast cancer-related mortality rates by 43.0% between 1989 and 2020 [2]. The 5-year breast cancer survival rate has also improved, contributing to a growing number of patients living with and beyond breast cancer [3,4]. Although encouraging, people living with and beyond breast cancer face numerous challenges throughout the breast cancer care continuum, including adverse events from highly invasive treatments [5,6,7], resulting in reduced ability to perform activities of daily living, work disability, and difficulties coping with psychological demands [8,9].

The World Health Organization (WHO) recognizes rehabilitation as a set of interventions that can help minimize the disabling effects of chronic conditions, such as cancer, by equipping people with self-management strategies or by providing services to optimize functioning [10]. Global estimates indicate a substantial and growing need for rehabilitation, driven largely by aging populations, the rising prevalence of chronic conditions, and injury-related morbidity [11]. Historically, however, rehabilitation has been conceptualized as a specialized service for individuals with disabilities rather than as an essential component of health systems, which has contributed to its limited prioritization in policy frameworks [12,13]. To address the increasing burden of rehabilitation, the WHO released the “Rehabilitation 2030: A call for action” [10,14], which is an initiative that draws attention to the global unmet need for rehabilitation and emphasizes that efforts should be directed towards supporting the health system in integrating rehabilitation into all levels of healthcare [10]. As part of this initiative, the WHO has set sustainable development goals, one of which is to promote well-being for all individuals at all ages and to reiterate the importance of healthy life expectancy [10,14]. In Canada, access to publicly funded rehabilitation services remains limited, with most care being delivered in private clinics and paid out-of-pocket by patients. This restricted accessibility underscores the fact that, despite being a high-income country, Canada still faces important challenges in ensuring equitable access to rehabilitation services.

Beyond the lack of rehabilitation services, a growing concern has been the underutilization of these services, even when they are accessible [15]. A qualitative study exploring the experiences of physical therapists (PTs) delivering rehabilitation in oncology settings revealed that PTs often felt that the benefits of physical therapy interventions and exercise were not fully understood by the multidisciplinary oncology team, resulting in fewer referrals for rehabilitation services [16,17,18]. The lack of continuity between breast cancer treatments and the management of treatment-related side effects through physical and psychological rehabilitation has also been identified by patients as a barrier to rehabilitation, causing women to feel abandoned by the healthcare system and contributing to increased anxiety and depressive symptoms [19,20]. In the province of Quebec, Canada, rehabilitation is generally not presented as an integral part of the oncology care pathway. Preoperative rehabilitation is rarely emphasized, and postoperative access to services tends to vary across regions. In many cases, information is limited to pamphlets with standardized exercises, which means that structured rehabilitation services are not systematically introduced or discussed with patients throughout the care trajectory [21].

While there is insufficient evidence to support a specific rehabilitation intervention for optimal postoperative recovery in breast cancer patients [22], the most recent cancer care guidelines endorsed the benefits of multimodal physical and psychological rehabilitation interventions in enhancing the well-being of women diagnosed with breast cancer, whether awaiting or undergoing treatments, or in remission [22,23,24,25]. However, a scoping review of perioperative rehabilitation interventions reported that up to 75.0% (MED = 9.0; IQR = 30.0) of breast cancer patients do not participate in rehabilitation interventions. Among those who engage, up to 58.0% (MED = 10.0; IQR = 12.8) drop out of their rehabilitation care [26], highlighting that several women’s needs remain unmet and that important barriers in the breast cancer care continuum limit participation and interest in rehabilitation [27]. Understanding women’s needs and expectations is crucial to providing tailored rehabilitation services and improving compliance [26].

Current evidence investigating breast cancer patients’ experiences with rehabilitation emerges mainly from small sample size studies conducted in homogenous settings, which may not provide a comprehensive portrait of women’s needs and rehabilitation opportunities. Multiple factors, including patients’ cultural capital (e.g., educational attainment, health literacy, past experiences, and communication competencies), social capital (e.g., connections and social network), distance from healthcare facilities, and clinical factors (e.g., tumor stage, adjuvant therapies), may explain the significant disparities patients report in their rehabilitation process [19]. Larger-scale studies addressing these differences could provide valuable insights for decision-makers in implementing provincial and national rehabilitation programs integrated within the breast cancer care continuum. Therefore, the aim of the present study was to investigate breast cancer patients’ rehabilitation needs and experiences and to document the barriers encountered at different time points along the breast cancer care continuum. This objective was divided into four specific objectives: (1) to describe sociodemographic and clinical profiles of patients who underwent mastectomy for breast cancer within the past five years; (2) to describe the rehabilitation needs expressed at each phase of the breast cancer care pathway, as well as the proportion of participants who utilized rehabilitation services, and barriers they encountered; (3) to compare sociodemographic and clinical characteristics between (a) participants who expressed rehabilitation needs and those who did not, (b) participants who used rehabilitation services and those who did not, and (c) participants who experienced specific barriers to rehabilitation and those who did not; and (4) to identify sociodemographic and clinical characteristics independently associated with (a) the timing of rehabilitation needs, (b) the use of rehabilitation services, and (c) the types of barriers encountered.

## 2. Methods

### 2.1. Study Design and Population

A cross-sectional study consisting of a self-reported online survey was conducted. This study and the questionnaire were reviewed and approved by the Université du Québec à Trois-Rivières (UQTR) Independent Research Ethics Board (CER-22-289-07.03).

Women aged 18 years and over with a diagnosis of breast cancer, who underwent breast surgery (i.e., conservative breast surgery, partial mastectomy, or total mastectomy) in the province of Quebec, Canada, were considered eligible for this study. To minimize the risk of recall bias [28], women were excluded if, at the time of administering the survey, more than 5 years had passed since their surgery. We aimed to recruit a total of 500 participants, and for the sample to be representative of different clinical and geographical realities, including women from all administrative regions with diverse socio-economic profiles. Women were recruited between 1 November 2022, and 1 September 2023 through sponsored advertisements published on the web pages and social media of the UQTR and the Fondation cancer du sein du Québec. These institutional and healthcare-related organizations promoted the study by providing an Internet link that directed potential participants to the online consent form. After providing consent, participants were asked two questions designed to ensure their eligibility for the survey before proceeding to the electronic questionnaire.

### 2.2. Survey Development

The questionnaire was initially developed by the research team members, drawing on the latest scientific evidence on perioperative rehabilitation interventions and clinical outcomes measures and their expertise in rehabilitation, healthcare services organization, and psychosociology. Rehabilitation needs were defined as aspects of functioning that may be affected following breast surgery, and that could impede women’s physical and psychosocial recovery as well as their participation in daily life. To ensure conceptual rigor, we based our framework on the International Classification of Functioning (ICF) Core Set for Breast Cancer to identify the components most likely to be impacted and relevant to post-mastectomy functioning and to the available resources and organizational context of the Quebec health system [9,29]. Content validity of the questionnaire was evaluated by an expert group including professionals in rehabilitation (i.e., PTs, kinesiologists), psychosociology, oncology care (i.e., a pivot nurse in oncology, a radiation oncologist, and a general surgeon), and two patient partners. Subsequently, face validity was established by two breast cancer patients who completed the questionnaire and provided feedback. A plain-language definition of rehabilitation, adapted from the World Health Organization and reviewed by our patient partners for clarity, was provided to participants: “Rehabilitation refers to a set of interventions or services that help people improve their ability to function in daily life and reduce the difficulties caused by a health condition, in interaction with their environment.” The list of physical and psychosocial interventions included in the survey was developed from the literature [26] and refined to reflect the structure of care and available rehabilitation resources in Quebec. The final questionnaire (Supplementary File S1) was divided into 4 sections: (1) Sociodemographic characteristics (14 questions); (2) Clinical profiles (12 questions); (3) Rehabilitation needs (5 questions); and (4) Rehabilitation experiences (6 questions). The census metropolitan influenced zone (MIZ) classification was used to further characterize participants’ geographic location by differentiating municipalities within urban and rural areas based on their degree of social and economic integration with urban cores [30,31]. Municipalities were assigned to the following categories: (0–1) No MIZ or Weak: Rural area with weak or no links to an urban core; (2) Moderate MIZ: Rural area with moderate links to an urban core; (3) Strong MIZ: Rural area with strong links with an urban core; (4) Census agglomeration (CA): Urban area with an urban core population between 10,000 and 100,000; (5) Census metropolitan area (CMA): Urban area with an urban core population of at least 100,000. The distance (km) between participants’ place of residence and the hospital center was also recorded to account for geographic accessibility. Rehabilitation needs and experiences were questioned at four time points along the continuum of care, which was divided into 4 phases: (i) Phase 1: from diagnosis to surgery; (ii) Phase 2: immediately after surgery; (iii) Phase 3: a few weeks after surgery or during complementary treatments; and (iv) Phase 4: a few weeks to a few months after the end of complementary treatments. The questionnaire included closed-ended questions (i.e., single- or multiple-choice questions or Likert scales), the results of which are reported in this paper. Two additional open-ended questions were included to inform the subsequent qualitative phase of this project.

### 2.3. Data Collection

The questionnaire was administered through the online platform Qualtrics *©* (Provo, UT, USA) and was completed once by each participant, lasting approximately 20 min. Written informed consent was obtained from all participants prior to participation.

### 2.4. Statistical Analysis

Descriptive statistics, namely frequencies, percentages, medians, means and standard deviations (SDs), were computed to describe the study sample, their rehabilitation needs, barriers, and use of rehabilitation services (specific objectives 1 and 2). To answer specific objective 3, bivariate analyses, using the chi-square test, were conducted to examine the differences between groups. Binomial logistic regression analyses were then conducted to examine, among variables that differed significantly between groups, those independently associated with the timing of rehabilitation needs, the use of rehabilitation services, and the types of barriers encountered (specific objective 4). Types of surgery (i.e., total mastectomy or partial mastectomy) and timing of complementary treatments (i.e., none, after surgery or before and after surgery) were included as covariates to control for potential confounding effects. Adjusted odds ratios and their 95% confidence intervals (CIs) are reported. All statistical analyses were performed using IBM SPSS Statistics for Windows, Version 18.0.0 (IBM Corp.: Armonk, NY, USA) and considered *p* values < 0.05 statistically significant.

## 3. Results

Five hundred and nineteen participants completed the questionnaire, of whom seventy-four (14.3%) only partially completed it. Demographic characteristics of respondents and partial respondents were comparable, with the exceptions of private insurance coverage and weekly physical activity levels. A significantly higher proportion of full respondents (74.6%) had private insurance drug coverage compared to partial respondents (62.5%) (*p* = 0.04). Additionally, partial respondents were more likely (32.4%) than full respondents (13.7%) to report no physical activity days per week on average (*p* = 0.003). Participants’ clinical profiles were similar, except that a higher proportion of full respondents reported receiving complementary treatments after surgery (61.8% vs. 28.4%) or a combination of treatments before and after surgery (26.7% vs. 10.8%), compared to partial respondents (*p* < 0.001).

### 3.1. Specific Objective 1

#### Participants’ Characteristics

Sociodemographic characteristics of participants are summarized in Table 1. The mean age at survey completion was 53.9 ± 10.5 years, compared to 51.6 ± 10.5 years at diagnosis. The sample included participants from all Quebec’s administrative regions, including 104 (20.0%) from Montérégie, 58 (11.2%) from Mauricie, 50 (9.6%) from Capitale-Nationale, and 48 (9.2%) from Montréal. Four hundred and five respondents (78.0%) were classified as living in a CA or CMA (urban areas), while 1.7% of the study sample was classified as living in a weak MIZ, 9.6% in a moderate MIZ, and 8.5% in a strong MIZ. Most respondents lived less than 10 km (45.7%) or less than 40 km (26.8%) from their healthcare facility. Less than 2.0% of the sample reported belonging to an indigenous community (1.2%) or a minoritized ethnic group (1.5%). Two hundred and forty-five respondents (47.2%) were living with another adult and one or more children, and three hundred and twenty-two participants (62.0%) were full-time workers at diagnosis. Most respondents (55.9%) had a college or an undergraduate degree at diagnosis.

Table 2 presents participants’ clinical profiles. The median delay between diagnosis and surgery was 55.00 days (IQR: 15.08). Two hundred and seventy-six participants (53.3%) were diagnosed with in situ or early-stage breast cancer, 36.6% with a locally advanced breast cancer, and 1.5% with a metastatic cancer. Thirty-six respondents (6.9%) reported their cancer was associated with an inherited genetic mutation. Over three-quarters (75.5%) of the sample underwent unilateral breast surgery, the most reported surgical procedure being the partial mastectomy (52.8%), followed by the total mastectomy (42.8%). Four hundred and thirty-eight participants (84.4%) received complementary treatments (i.e., systemic treatments, radiation therapy, hormonal or endocrine therapy), most of which were administered after surgery (67.6%).

### 3.2. Specific Objective 2

#### 3.2.1. Rehabilitation Needs

**Figure 1:**
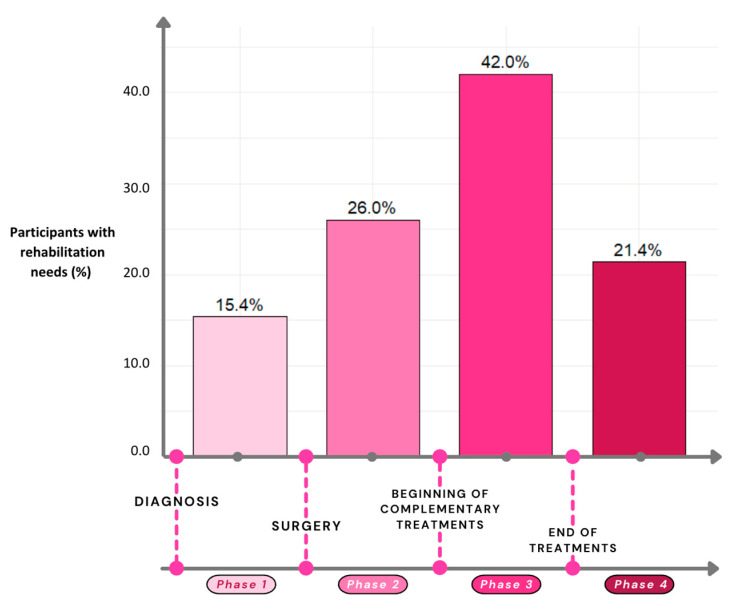
Rehabilitation needs at different time points of the breast cancer care continuum.

Figure 1 illustrates the percentage of participants who expressed rehabilitation needs at each phase of the breast cancer care continuum. Two hundred and eighteen participants (42.0%) expressed rehabilitation needs a few weeks after surgery or during complementary treatments (Phase 3), while only 15.4% of the sample had rehabilitation needs in the period between diagnosis and surgery (Phase 1). One hundred and twelve participants (21.6%) reported no rehabilitation needs throughout their care pathway. Obtaining resources to promote faster recovery from surgery, preventing loss of functional capacities, and being informed and reassured about available rehabilitation services were the most reported needs prior to surgery, expressed as very or extremely important by 66.3%, 62.6% and 62.5% of the participants, respectively. As for rehabilitation needs during or after breast cancer treatments, improving physical condition, preventing loss of functional capacities, and obtaining resources to promote faster recovery from surgery were identified as very or extremely important by 46.7%, 41.5% and 38.3% of the participants, respectively. Supplementary File S2 details rehabilitation needs at each phase of the breast cancer care continuum and the extent to which they were considered important by participants.

**Figure 2:**
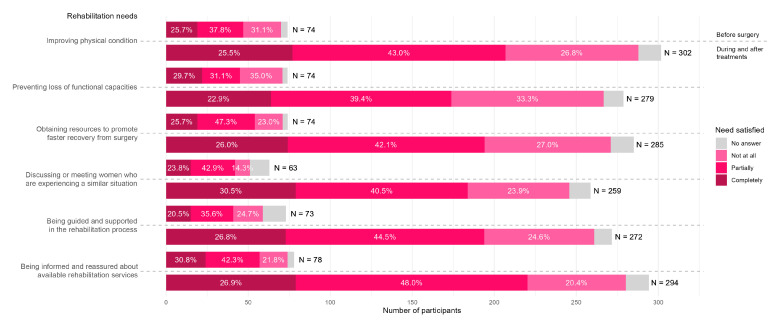
Perioperative rehabilitation needs and their perceived adequacy.

Figure 2 illustrates the extent to which rehabilitation needs were met before and after surgery. For each rehabilitation need expressed before surgery, 14.3% to 35.1% of the participants felt their needs were not met at all, 31.1% to 47.3% felt their needs were only partially met, and 20.5% to 29.7% felt their rehabilitation needs were completely met. The need “obtaining resources to promote faster recovery from surgery” was the least satisfied before surgery. For the period during and after treatments, 20.4% to 33.0% of the participants felt their needs were not met at all, 39.4% to 48.0% felt their needs were only partially met, and between 22.9% and 30.5% felt their rehabilitation needs were completely met. The need “preventing loss of functional capacities” was the least satisfied during and after treatments.

#### 3.2.2. Rehabilitation Experiences

**Figure 3:**
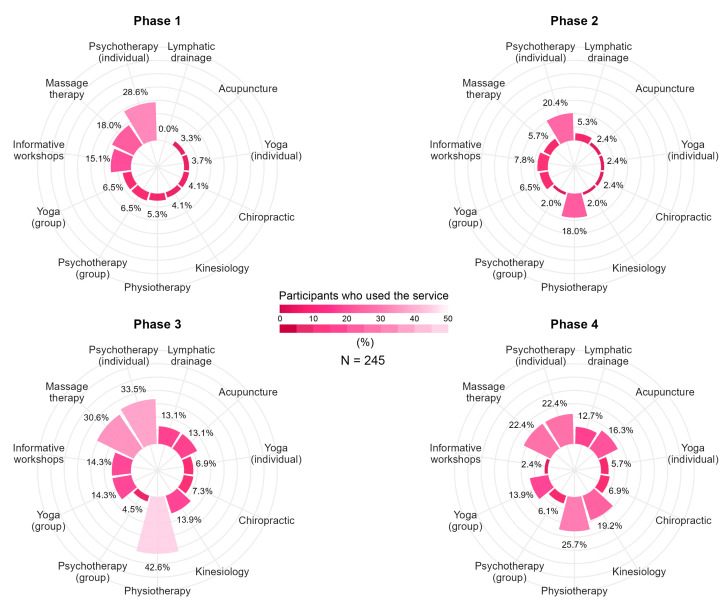
Rehabilitation service use at different time points of the breast cancer care continuum.

Two hundred and forty-five participants (47.2%) reported the use of rehabilitation services throughout their care pathway, the most common being physiotherapy (68.1%), psychotherapy (60.4%) and massage therapy (52.7%). The percentage of participants using rehabilitation services varied throughout the continuum of care. Most of the participants (51.8%) reported using rehabilitation services in Phase 1, compared to 46.5% in the period immediately after surgery (Phase 2). In Phase 3, 78.0% of the sample reported using such services, and 60.0% did so after completing complementary treatments (Phase 4). Figure 3 presents, for each service, the percentage of participants who used a given service at each phase of the breast cancer care continuum.

#### 3.2.3. Barriers When Using Rehabilitation Services

One hundred and seventy-five participants (71.4%) reported facing barriers when using rehabilitation services, the most common being financial constraints (27.3%), impaired physical function (26.1%), and loss of motivation (22.0%). Among participants who did use rehabilitation services, 94.3% mentioned they would have liked to access at least one other service, with massage therapy (22.4%), informative workshops (21.6%), and individual yoga sessions (20.0%) being the most frequently reported. Not being aware of the availability of the service (28.8%), the fact that the service was not prescribed or recommended (32.6%), and financial constraints (20.8%) were the most reported barriers to service utilization.

#### 3.2.4. Barriers to Participation

As for participants who did not use rehabilitation services, 75.5% of them would have liked to have access to rehabilitation services. Massage therapy (42.3%), physiotherapy (33.7%) and individual psychotherapy sessions (33.2%) were the most desired services for this subgroup. The most frequently reported barriers to accessing services were a lack of awareness of the service’s availability (41.7%), the fact that the service was not prescribed or recommended (38.3%), and financial constraints (16.7%). Supplementary File S3 presents all reported barriers to service utilization and desired rehabilitation services for both participants who accessed services and those who did not.

### 3.3. Specific Objective 3

#### 3.3.1. Differences in Patient Profile and the Timing of Rehabilitation Needs

Differences in sociodemographic and clinical characteristics were found between women expressing rehabilitation needs and those who did not throughout the breast cancer care continuum.

Before surgery (Phase 1), a significantly higher proportion of participants with a graduate degree expressed rehabilitation needs (32.9%) compared with those whose highest level of education was a college degree (12.8%) or an undergraduate degree (15.2%) (*p* = 0.001). In addition, a significantly higher proportion of participants with an annual income between CAD 50,000 and 89,999 reported rehabilitation needs prior to surgery compared with those earning less than CAD 49,999 a year (*p* = 0.03).

Participants expressing rehabilitation needs immediately after surgery (Phase 2) were comparable to those who did not, except for the timing of complementary treatments. Participants who received complementary treatments both before and after surgery reported rehabilitation needs more frequently in phase 2 compared to participants who received treatments only after surgery (38.7% vs. 26.2%; *p* = 0.04).

A few weeks after surgery or during adjuvant treatments (Phase 3), rehabilitation needs were more frequently reported among the younger age group (18–35 years) compared to participants aged 65 years and older (65.6% vs. 34.8%; *p* = 0.008), and among women with a graduate degree (61.8%) compared with those holding a high school diploma (30.9%) or a college degree (42.5%) (*p* < 0.001). Significant differences in rehabilitation needs were also observed between women who underwent a total mastectomy compared to a partial mastectomy (57.3% vs. 39.2%; *p* < 0.001), between those who underwent bilateral and unilateral breast surgery (62.0% vs. 44.9%; *p* = 0.006), between women who had axillary lymph node dissection and those who did not (58.2% vs. 43.4%; *p* = 0.005), and between participants who underwent reconstructive surgery and those who did not (*p* = 0.002). Finally, rehabilitation needs were more frequently reported among participants who received complementary treatments both before and after surgery compared to those who received treatments only after surgery (63.7% vs. 41.6%; *p* < 0.001).

Following the completion of treatments (Phase 4), a higher proportion of women aged 36–50 years reported rehabilitation needs compared to those aged 65 years and older (31.0% vs. 10.9%; *p* = 0.021). Although the overall proportion of expressed needs decreased at this time point, significant differences persisted between participants who underwent axillary lymph node dissection and those who did not (30.6% vs. 20.2%; *p* = 0.021), as well as between participants who received complementary treatments both before and after surgery (30.6%) or only after surgery (25.2%) compared with those who did not receive complementary treatments (*p* = 0.001).

#### 3.3.2. Comparison of Patient Profiles Between Users and Non-Users of Rehabilitation Services

The sociodemographic characteristics of participants who used rehabilitation services and those who did not were different on several fronts. A higher proportion of participants in the 18–35 (70.0%) and 36–50 (62.4%) age groups used rehabilitation services compared with participants aged 51–64 (51.2%) and 65 years and older (41.9%) (*p* = 0.015). Similarly, participants with an undergraduate (65.0%) or graduate degree (68.5%) reported more frequently having used rehabilitation services than those with a high school diploma (28.3%) or a college degree (51.1%) (*p* < 0.001). Participants residing in a census agglomeration (66.2%) used rehabilitation services more frequently than those in rural areas (MIZ 0–1: 25.0%; MIZ 2: 39.5%) (*p* = 0.005). Finally, significantly higher proportions of participants with prescription drug coverage (62.7% vs. 39.4%) and private insurance for healthcare services (63.1% vs. 41.3%) used rehabilitation services (*p* < 0.001).

The clinical profiles of participants who used rehabilitation services and those who did not also showed significant differences. A greater proportion of participants with locally advanced breast cancer (64.2%) used rehabilitation services compared with those with early-stage disease (50.4%, *p* = 0.008). Use of rehabilitation services was higher among participants who underwent a total mastectomy (61.7% vs. 52.3%, *p* = 0.015), had an axillary lymph node dissection (65.9% vs. 52.3%, *p* = 0.011), or received complementary treatments both before and after surgery (72.0%), compared with those who received treatments only after surgery (53.4%) or no complementary treatments (28.6%, *p* < 0.001). In contrast, undergoing reconstructive surgery did not appear to influence rehabilitation service use (*p* ≥ 0.05).

#### 3.3.3. Sociodemographic Disparities in Participants Reporting Barriers to Rehabilitation

Significant differences were observed in the distribution of specific barriers according to participants’ sociodemographic characteristics. Participants aged 18–35 years reported more frequently that the rehabilitation service did not meet their needs (18.2% vs. 2.4%; *p* < 0.001), that they experienced time constraints (22.7% vs. 3.6%; *p* = 0.01), and reduced psychological capacity (27.3% vs. 6.0%; *p* = 0.018) than those aged 50–64 years. Younger participants also more frequently indicated that the lack of prescription or recommendation contributed to non-use of services (66.7% vs. 35.8% among participants aged 51–64 years and 33.3% among those aged 65 years and older; *p* = 0.007).

Transportation barriers were reported more often by participants with lower socioeconomic status. They were more frequent among participants with annual incomes below CAD 50,000 compared with those earning above CAD 90,000 (14.0% vs. 3.0%; *p* = 0.013), among those with a high school diploma or less compared to women with higher educational levels (27.8%; *p* = 0.04), and among women living alone (33.3% vs. 7.6%; *p* = 0.011) or single mothers (9.5% vs. 1.4%; *p* = 0.036) compared to those living with a partner.

Financial constraints preventing rehabilitation service use were more frequently reported by participants with annual incomes below CAD 50,000 compared with those earning above CAD 90,000 (40.0% vs. 17.0%; *p* = 0.007) and more often by women living alone compared to those living with a partner. (44.4% vs. 20.4%; *p* = 0.03).

#### 3.3.4. Clinical Differences in Participants Reporting Barriers to Rehabilitation

Differences in reported barriers were also observed according to clinical characteristics. Participants diagnosed with locally advanced (17.0%) or metastatic breast cancer (40.0%) report more frequently that the rehabilitation services they used did not meet their needs compared with those with early-stage cancer (7.0%; *p* = 0.010). Moreover, a higher proportion of participants with metastatic disease reported lacking the physical capacity to sustain participation in a rehabilitation program (80.0%; *p* = 0.020). Significantly higher proportions of participants who underwent bilateral breast surgery (20.8% vs. 9.6%; *p* = 0.027) or total mastectomy (16.9% vs. 6.7%; *p* = 0.013) reported that the rehabilitation services used did not adequately meet their needs. Conversely, participants who underwent reconstructive surgery did not report distinct rehabilitation needs compared with those who did not (*p* ≥ 0.05).

### 3.4. Specific Objective 4

The following subsections present the sociodemographic and clinical characteristics as well as their associations with one or more of the following outcomes: timing of rehabilitation needs, use of rehabilitation services, and barriers to rehabilitation.

#### 3.4.1. Age

No significant associations were found between age groups and either the timing of rehabilitation needs or the use of rehabilitation services at any phase of the breast cancer continuum (all *p* values ≥ 0.05). Compared with the younger age group (18–35 years), participants aged 50–64 years were less likely to report unmet needs after using rehabilitation services (adjusted OR = 0.12, 95% CI [0.02–0.72]; *p* = 0.02), to experience time constraints (adjusted OR = 0.13, 95% CI [0.03–0.59]; *p* = 0.008), or to report impaired participation due to limited psychological capacity (adjusted OR = 0.15, 95% CI [0.04–0.57]; *p* = 0.006). Participants aged 65 years and older were significantly less likely to report financial constraints compared to the younger age group (adjusted OR 0.09, 95% CI [0.01–0.80]; *p* = 0.03).

#### 3.4.2. Geographic Location

Neither rural/urban classification nor distance from healthcare facility was associated with the timing of rehabilitation needs (all *p* values ≥ 0.05). However, participants classified as residing in MIZ 2 rural areas were 54% less likely to report having used rehabilitation services compared with those living in a CMA (adjusted OR 0.46; 95% CI [0.23–0.91]; *p* = 0.03). Living in MIZ 3 rural areas (adjusted OR 5.82, 95% CI [1.52–22.3]; *p* = 0.01), between 41 and 100 km from the healthcare facility (adjusted OR 12.23, 95% CI [1.2–124.45]; *p* = 0.03) or more than 100 km (adjusted OR 25.24, 95% CI [2.36–270.12]; *p* = 0.008) were associated with higher odds of experiencing transportation barriers compared with those living in a CMA. Residing in MIZ 0–1 areas (adjusted OR = 6.48, 95% CI [1.14–36.91]; *p* = 0.04) or living more than 100 km from a healthcare facility (adjusted OR = 2.75, 95% CI [1.09–6.95]; *p* = 0.03) were associated with significantly higher odds of not engaging in rehabilitation services due to financial constraints. Additionally, participants living more than 100 km from their healthcare facility were significantly more likely to have not engaged in rehabilitation interventions due to excessive delays in accessing services (OR = 4.49, 95% CI [1.43–14.16]; *p* = 0.01).

#### 3.4.3. Income

Before surgery (Phase 1), participants with annual household incomes of CAD 50,000–89,999 had higher odds of expressing rehabilitation needs compared to those earning less than CAD 49,999 (adjusted OR 2.68, 95% CI [1.17–6.11]; *p* = 0.02). The use of rehabilitation services was not associated with annual household incomes (all *p* values ≥ 0.05). However, participants with prescription drug coverage (adjusted OR 2.59; 95% CI [1.63–4.10]; *p* < 0.001) or private insurance for healthcare services (adjusted OR 2.40; 95% CI [1.56–3.69]; *p* < 0.001) were more likely to report having used rehabilitation services compared with participants without private insurance. Compared to participants reporting annual household incomes below CAD 49,999, participants earning between CAD 50,000–89,999 (adjusted OR 0.44, 95% CI [0.20–0.98]; *p* = 0.04) or above CAD 90,000 (adjusted OR 0.26, 95% CI [0.12–0.57]; *p* < 0.001) were significantly less likely to have been unable to engage in rehabilitation interventions due to financial constraints. Furthermore, participants reporting annual household incomes above CAD 90,000 were significantly less likely to have encountered transportation barriers compared with those earning less than CAD 49,999 (adjusted OR 0.19, 95% CI [0.05–0.82]; *p* = 0.03).

#### 3.4.4. Education

No associations between the highest level of educational attainment and the timing of rehabilitation needs were observed at any phase of the breast cancer care continuum, except a few weeks after surgery or during treatments (Phase 3). At this time point, participants with an undergraduate degree (adjusted OR = 2.70, 95% CI [1.37–5.34]; *p* = 0.004) and those with a graduate degree (adjusted OR = 3.63, 95% CI [1.70–7.74]; *p* < 0.001) were significantly more likely to report rehabilitation needs compared with participants whose highest education level was a high school diploma or less. Similarly, higher education levels were associated with greater use of rehabilitation services, with participants holding a college degree (adjusted OR = 2.78, 95% CI [1.39–5.55]; *p* = 0.004), an undergraduate degree (adjusted OR = 4.82, 95% CI [2.36–9.87]; *p* < 0.001), or a graduate degree (adjusted OR = 6.10, 95% CI [2.71–13.70]; *p* < 0.001) being increasingly more likely to report having used rehabilitation services compared with participants with a high school diploma or less. Participants whose highest level of education was a high school diploma faced significantly more transportation barriers related to rehabilitation services compared with those with a graduate degree (adjusted OR = 5.79, 95% CI [1.20–27.84]; *p* = 0.03).

#### 3.4.5. Household Composition at Diagnosis

No associations were found between household composition and the timing of rehabilitation needs, and the use of rehabilitation services (all *p* values ≥ 0.05). Nevertheless, participants living with a partner and children (OR = 0.29, 95% CI [0.13–0.64]; *p* = 0.002) or with a partner only (OR = 0.38, 95% CI [0.16–0.91]; *p* = 0.03) were significantly less likely to have experienced financial barriers that prevented them from engaging in rehabilitation interventions compared to participants living alone. No significant associations were found between household composition at diagnosis and the likelihood of experiencing transportation barriers (*p* ≥ 0.05).

#### 3.4.6. Type of Surgery and Cancer Type

The type of surgery was independently associated with rehabilitation needs a few weeks after surgery and during treatments (Phase 3). At this time point, patients who underwent total mastectomy had 75% higher odds of reporting rehabilitation needs compared with those who underwent partial mastectomy (adjusted OR 1.75 [1.18–2.59]; *p* = 0.005). Furthermore, compared with participants who underwent partial mastectomy, those who had a total mastectomy were 51% more likely to report having used rehabilitation services (adjusted OR = 1.51, 95% CI [1.10–2.28]; *p* = 0.04) and almost three times as likely to report unmet rehabilitation needs (adjusted OR = 2.81, 95% CI [1.17–6.74]; *p* = 0.02).

Although significant differences in rehabilitation barriers were observed between groups with different cancer types, this variable was not independently associated with any specific rehabilitation barriers (all *p* values ≥ 0.05).

#### 3.4.7. Timing of Complementary Treatments

No associations between the timing of complementary treatments and rehabilitation needs were observed at any phase of the breast cancer care continuum, except after treatment completion (Phase 4) where participants who received complementary treatments either after surgery (adjusted OR 12.23, 95% CI [1.64–91.0]; *p* = 0.01) or both before and after surgery (adjusted OR 17.72, 95% CI [2.35–133.78]; *p* = 0.005) were significantly more likely to report rehabilitation needs compared with those who did not receive complementary treatments. A similar pattern was observed in rehabilitation service utilization, with participants who received complementary treatments after surgery exhibiting threefold higher odds of service use (adjusted OR 3.18, 95% CI [1.53–6.61]; *p* = 0.002), and those receiving treatments both before and after surgery showing sixfold higher odds (adjusted OR 6.38, 95% CI [2.90–14.03]; *p* < 0.001), compared with participants who did not receive complementary treatments. The timing of complementary treatments was not independently associated with any specific rehabilitation barriers (all *p* values ≥ 0.05).

## 4. Discussion

The aim of this study was to obtain a better understanding of rehabilitation needs and experiences of care of women diagnosed with breast cancer who had undergone breast surgery, and to document the barriers to rehabilitation encountered at different time points of the breast cancer care continuum, and to examine the influence of sociodemographic and clinical factors on these outcomes.

The study sample included over five hundred respondents (mean age: 53.9 years), with participants from all 17 administrative regions of the Quebec province. Most participants were aged 36–50 or 51–64 years, reflecting the age distribution associated with higher breast cancer prevalence. Notably, two of the three administrative regions most represented in this study (Montérégie and Montréal) have over 85.0% of their population residing in urban centers [30]. Furthermore, almost 90.0% of survey respondents reported living in urban areas or in rural areas closely connected to an urban core. Despite being representative of the distribution of Quebec’s population in urban and rural areas, the perspectives of patients living in remote rural areas (MIZ 0 to 2) may be under-represented in the results of this survey, given the small number of participants from these regions. As access to health resources may pose an additional challenge in these areas, exploring their rehabilitation experiences in greater detail seems necessary. Moreover, less than 2.0% of respondents belonged to a minoritized ethnic group or to an indigenous community, which is lower than the proportions reported in the latest census of Quebec’s population [31]. Indeed, in 2021, 14.9% of Quebec residents aged 15 and over were members of a visible minority, and 2.5% identified themselves as members of an indigenous community [31]. As race, ethnicity and cultural identity are factors known to impact health needs and to contribute to health inequalities in access to care [32,33,34], it is conceivable that the rehabilitation experiences of these groups may differ and may not have been fully captured by the results of this survey.

Most participants included in this study were diagnosed with early-stage breast cancer and underwent unilateral breast surgery, with the most frequently reported surgical procedure being the partial mastectomy. Although the diagnosis of breast cancer remains a physically and emotionally challenging experience, irrespective of cancer type, treatment of early-stage breast cancers generally involves less invasive surgical procedures with fewer side effects [7], which can result in less extensive needs for rehabilitation. Our study results show that participants who underwent total mastectomy were significantly more likely to report rehabilitation needs in the postoperative period, which translated into higher use of rehabilitation services. However, these women were also more likely to report that the services they received did not adequately meet their needs, suggesting that current rehabilitation interventions may not be sufficiently tailored to patients undergoing more complex or intensive treatments. Moreover, receiving complementary treatments was associated with persistent rehabilitation needs after treatment completion and significantly increased the likelihood of utilizing rehabilitation services. These associations might be partly explained by musculoskeletal adverse events often arising from combined surgical and complementary treatments [7,35], which can impair upper limb function and limit activity participation, resulting in greater needs for rehabilitation.

Nearly 80.0% of respondents expressed rehabilitation needs at some point of the breast cancer care continuum, with the highest demand occurring a few weeks after surgery. This finding was especially significant for patients who had reconstructive surgery, as they similarly reported a higher proportion of rehabilitation needs in the postoperative period. However, less than 50.0% of participants reported the use of rehabilitation services. Despite several international clinical practice guidelines stressing the importance of rehabilitation in the cancer care continuum [22,25,36,37,38,39], from diagnosis to the end of treatments, the under-utilization of rehabilitation services undoubtedly reflects barriers to utilization. In a call to action published following a roundtable meeting of the American Cancer Society, Schmitz et al. [40] highlighted the disconnection between oncology, surgery and rehabilitation services as a major challenge in cancer rehabilitation. This issue was further reiterated in the qualitative studies by Rafn et al. [19] and Brennan et al. [16], where PTs described unstructured referral pathways and poor connections between acute and community services as factors hindering oncology professionals from referring to rehabilitation services. Lack of awareness of the role and benefits of rehabilitation among both oncology experts and breast cancer patients has also been reported, with patients pointing out that education about rehabilitation was never felt as a priority, leaving them uncertain and concerned about whether rehabilitation services would be helpful or harmful to their condition [15,16,19,40]. These findings are consistent with the results of the present survey, which highlighted that the needs for information about available rehabilitation services and guidance through the rehabilitation process were among the least satisfied. The lack of awareness of rehabilitation services, combined with the fragmented integration of these services into tertiary care cancer programs [15,41], could also explain the lower utilization rates before and immediately after surgery, while oncology protocols were still being implemented.

The physical and emotional burden associated with the diagnosis and treatment of breast cancer, which may have prevented patients from using rehabilitation services, must also be considered. Young breast cancer patients (i.e., 18–50 years) from Atlantic Canada who participated in telephone interviews identified their desires to resume a normal life, to minimize medical appointments, and to prioritize their families over sacrificing personal time and financial resources as personal barriers to rehabilitation in the postoperative period [41]. Although no associations were found between service utilization and age, our study findings revealed that participants aged 18–35 years were more likely to express dissatisfaction, reporting that rehabilitation services did not meet their needs or failed to accommodate their time constraints or psychological burden. Furthermore, they were more likely to face financial constraints compared to participants aged 65 years and older. These challenges appeared to be further compounded for single women, who experienced greater financial constraints compared with those living with a partner. These results underscore the importance of developing age-sensitive and context-specific rehabilitation interventions that consider the competing personal, professional, and financial demands faced by individuals throughout the breast cancer care continuum, with younger women navigating survivorship being particularly affected.

A scoping review investigating perioperative rehabilitation interventions and experiences of breast cancer patients also revealed significant barriers to participation and completion of rehabilitation programs [26]. Transportation issues, lack of motivation, and financial constraints were cited as major barriers to adherence. These findings raise concerns regarding structural inequities in access to rehabilitation, suggesting that socioeconomic and geographic factors such as income, insurance coverage, and residential location may substantially influence engagement in rehabilitation interventions and continuity of care. Consistent with these observations, our results showed that participants without private insurance coverage, with lower household incomes, or with lower educational attainment were more likely to have faced financial and transportation barriers that hindered their engagement in rehabilitation services. Geographic inequities were also evident, as women residing in rural areas or at considerable distance from their healthcare facility were significantly more likely to have experienced excessive delays in accessing services and transportation barriers than those living closer to urban centers. Together, these findings point to persistent inequities in rehabilitation care and underscore how social determinants of health continue to shape access to and participation in cancer rehabilitation.

These disparities call for concrete actions to enhance accessibility and ensure that all women can fully benefit from rehabilitation services throughout the breast cancer care continuum. Integrating supportive mechanisms within oncology care pathways, such as expanding publicly funded rehabilitation services, improving transportation support, and offering home-based rehabilitation interventions, could mitigate the impact of socioeconomic vulnerability, ultimately promoting more equitable recovery trajectories for women with breast cancer.

### 4.1. Strengths and Limitations

While this study benefits from a broad geographic reach across the province of Quebec, its findings might not be applicable to other provinces and countries. The online survey format facilitates ease of access and participation. However, the study has potential selection bias, as it may primarily attract respondents who are more digitally literate or actively engaged with their healthcare. In addition, the survey was conducted only in French, which may have excluded non-French-speaking participants, thus limiting the diversity of perspectives. Incomplete responses from 74 participants may have also impacted the completeness of the dataset and should be considered when interpreting the results. Furthermore, although the questionnaire was grounded in existing evidence and expert consultation, the lack of a conceptual framework may have limited its ability to fully capture the multidimensionality of healthcare needs. Future studies would benefit from integrating emerging frameworks, such as the NEED framework, to guide item selection and ensure comprehensive assessment of relevant domains [42]. Additionally, although participants who underwent breast surgery more than 5 years prior to survey completion were excluded, there remains a possibility of recall bias. Patients were asked to recall specific and complex aspects of their care continuum, which may have led to inaccuracies in their responses. Finally, some associations observed in this study were characterized by wide confidence intervals, suggesting variability in the estimates and warranting cautious interpretation of their precision and clinical significance.

### 4.2. Future Research Directions

This study highlights significant needs for rehabilitation services among women diagnosed with breast cancer, but demonstrates low utilization rates, reflecting systemic and personal barriers. Future research should strive to question a more representative sample of the population, including members of underserved communities, indigenous communities, and minoritized ethnic groups. Addressing the fragmented integration of rehabilitation services into the breast cancer care continuum should be prioritized, as both healthcare professionals and breast cancer patients perceive the lack of continuity between services as a major barrier. Additionally, it is crucial to address disparities in rehabilitation experiences across different sociodemographic groups, as women living alone, in rural areas, or with lower socioeconomic status were found to be more likely to face constraints limiting their access to rehabilitation services.

## 5. Conclusions

The breast cancer care continuum is associated with a physical and emotional burden resulting in significant needs for rehabilitation. The current structure of the healthcare system seems to hinder access to rehabilitation services, as the fragmented integration of these services into oncology care protocols contributes to a lack of awareness of the benefits of rehabilitation among both healthcare providers and breast cancer patients. Disparities in rehabilitation experiences based on sociodemographic factors, such as ethnicity, income, and geographic location, need to be further investigated to improve the rehabilitation services available to patients with breast cancer and to reduce inequalities in access to care and health outcomes.

## Figures and Tables

**Table 1:** Sociodemographic characteristics.

Sociodemographic Characteristics (N = 519)	Mean ± SD or N (%)	Missing Values N (%)
Age at survey completion (years)	53.9 ± 10.5	17 (3.3)
Age at diagnosis (year)	51.7 ± 10.5	47 (9.1)
**Age group—at diagnosis (years)**		
18–35	33 (6.4)	47 (9.1)
36–50	198 (38.2)	
51–64	190 (36.6)	
65 years and older	51 (9.8)	
**Administrative region**		
Bas St-Laurent	20 (3.9)	10 (1.9)
Saguenay Lac St-Jean	33 (6.4)	
Capitale-Nationale	50 (9.6)	
Mauricie	58 (11.2)	
Estrie	20 (3.9)	
Montréal	48 (9.2)	
Outaouais	22 (4.2)	
Abitibi-Témiscamingue	11 (2.1)	
Côte Nord	11 (2.1)	
Nord du Québec	1 (0.2)	
Gaspésie-Îles-de-la-Madeleine	6 (1.2)	
Chaudière-Appalaches	29 (5.6)	
Laval	19 (3.7)	
Lanaudière	22 (4.2)	
Laurentides	33 (6.4)	
Montérégie	104 (20.0)	
Centre-du-Québec	22 (4.2)	
**Classification of urban and rural areas**		
MIZ 0–1	9 (1.7)	11 (2.1)
MIZ 2	50 (9.6)	
MIZ 3	44 (8.5)	
CA	76 (14.6)	
CMA	329 (63.4)	
**Distance from healthcare facility (km)**		
<10 km	237 (45.7)	40 (7.7)
11–40 km	139 (26.8)	
41–100 km	63 (12.1)	
>100 km	40 (7.7)	
**Race and ethnicity**		
Belonging to an indigenous community (yes)	6 (1.2)	4 (0.8)
Belonging to an ethnic minority group (yes)	8 (1.5)	3 (0.6)
**Religion**		
Atheism	140 (27.0)	1 (0.2)
Buddhism	1 (0.2)	
Christianism	288 (55.5)	
Jewish	2 (0.4)	
Muslim	1 (0.2)	
Other	26 (11.6)	
Prefers not to answer	60 (11.4)	
**Household composition at diagnosis**		
One adult	72 (13.9)	6 (1.2)
One adult with one or more children	71 (13.7)	
Two adults	125 (24.1)	
Two adults with one or more children	245 (47.2)	
Had children under 18 years of age at diagnosis (yes)	174 (33.5)	12 (2.3)
**Social and familial support at diagnosis**		
More than expected	181 (34.9)	5 (1.0)
As much as expected	235 (45.3)	
Less than expected	98 (18.9)	
**Occupation at diagnosis**		
Full-time worker	322 (62.0)	6 (1.2)
Part-time worker	39 (7.5)	
Student	3 (0.6)	
Retired	104 (20.0)	
Unemployed	16 (3.1)	
Actively seeking employment	2 (0.4)	
Temporary sick leave	20 (3.9)	
Employment insurance beneficiary	13 (2.5)	
Welfare beneficiary	3 (0.6)	
**Highest educational attainment**		
Elementary school education	3 (0.6)	18 (3.5)
Secondary (high school) diploma	60 (11.6)	
College degree (pre-university or technical diploma)	134 (25.8)	
Trade school education (technical or occupational certificate)	67 (12.9)	
Undergraduate degree (bachelor’s or advanced diploma)	156 (30.1)	
Master’s degree (graduate education)	73 (14.1)	
Doctoral or post-doctoral degree (graduate education)	8 (1.5)	
**Annual household income at diagnosis ($ CAD)**		
<10,000	2 (0.4)	30 (5.8)
10,000–29,999	19 (3.7)	
30,000–49,999	74 (14.3)	
50,000–69,999	65 (12.5)	
70,000–89,999	55 (10.6)	
90,000–109,999	58 (11.2)	
110,000–129,999	42 (8.1)	
130,000–149,999	20 (3.9)	
150,000–169,999	23 (4.4)	
170,000–189,999	17 (3.3)	
190,000–199,999	3 (0.6)	
≥200,000	23 (4.4)	
Prefers not to answer	88 (17.0)	
**Private insurance coverage**		
Prescription drugs	361 (69.6)	26 (5.0)
Imaging	228 (43.9)	21 (4.0)
Privately funded healthcare	326 (62.8)	22 (4.2)
**Use of private insurances**		
Prescription drugs	361 (69.6)	-
Imaging	203 (12.9)	
Privately funded healthcare	227 (43.7)	
**Weekly physical activity (mean days)**		
0	85 (16.4)	-
1	67 (12.9)	
2	79 (15.2)	
3	103 (19.8)	
4	75 (14.5)	
5	71 (13.7)	
6	20 (3.9)	
7	19 (3.7)	

CA: Census agglomeration; CAD: Canadian dollar; CMA: Census metropolitan area; MIZ: Metropolitan influenced zone; SD: Standard deviation.

**Table 2:** Clinical profiles.

Clinical Characteristics (N = 519)	Mean ± SD or N (%)	Missing Values N (%)
Delay diagnosis-surgery (days)	Med: 55.0 IQR: 15.1	55 (10.5)
**Breast cancer type**		
In situ or early stage	276 (53.2)	29 (5.6)
Locally advanced	190 (36.6)	
Metastatic	8 (1.5)	
Do not know	16 (3.1)	
Breast cancer associated with inherited gene mutations (yes)	36 (6.9)	28 (5.4)
**Breast surgery**		
Unilateral	392 (75.5)	48 (9.2)
Bilateral	79 (15.2)	
Total mastectomy	222 (42.8)	
Partial mastectomy	274 (52.8)	
Immediate reconstruction	125 (24.1)	
Delayed reconstruction	56 (10.8)	
**Lymph node dissection**		
Sentinel lymph node dissection	375 (72.3)	48 (9.2)
Axillary lymph node dissection	137 (26.4)	
**Complementary treatments**		
Systemic treatments	244 (47.0)	48 (9.2)
Local radiation therapy	144 (27.7)	
Regional radiation therapy	208 (40.1)	
Hormonotherapy	286 (55.1)	
None	33 (6.4)	
**Timing of complementary treatments (N = 438)**		
Adjuvant treatments (after surgery)	296 (67.6)	10 (2.3)
Neoadjuvant and adjuvant treatments (before and after surgery)	127 (29.0)	
Do not know	5 (1.1)	

IQR: Interquartile range; Med: Median; SD: Standard deviation.

## Data Availability

All data is contained within the manuscript and the Supplementary Files. Further inquiries can be directed to the corresponding author.

## Supplementary Materials

The following supporting information can be downloaded at https://www.mdpi.com/article/10.3390/healthcare13243239/s1. File S1: Questionnaire; File S2: Rehabilitation needs; File S3: Barriers to rehabilitation.

## Abbreviations

The following abbreviations are used in this manuscript:

CA	Census agglomeration
CMA	Census metropolitan area
IQR	Interquartile range
MED	Median
MIZ	Metropolitan influenced zone
PTs	Physical therapists
SD	Standard deviation
WHO	World Health Organization
