# Supplementary material for: A Provincial Survey on the Perioperative Rehabilitation Needs and Experiences of Women Diagnosed with Breast Cancer
Source: Healthcare (Basel). 2025 Dec 10;13(24):3239. doi: 10.3390/healthcare13243239 (PMC12733279; doi:10.3390/healthcare13243239)
Supplement: Supplementary file 1 [file healthcare-13-03239-s001.zip › SupplementaryFile S1.pdf]

ELIGIBILITY CRITERIA

1. **Are you 18 years or older?**
   - o Yes
   - o No
2. **Have you undergone surgery (conservative, partial, or total mastectomy) for breast cancer in the last five years?**
   - o Yes
   - o No
3. **Was your surgery performed in Quebec, Canada?**
   - o Yes
   - o No

SECTION 1: SOCIO-DEMOGRAPHIC DATA

This first section will allow us to identify the socio-demographic characteristics of women who have undergone a mastectomy for breast cancer in the past five years. Please answer to the best of your knowledge.

1. **Personal Information**
   - o Date of birth________________
   - o City of residence _____________
2. **Do you consider yourself an Indigenous person (e.g., Inuit, Métis, or a member of First Nations)?**
   - o Yes
   - o No
   - o Prefer not to answer
3. **Do you consider yourself a racialized or ethnicized person or a member of a visible minority (i.e., a non-Indigenous person who is not white or does not have white skin)?**
   - o Yes
   - o No
   - o Prefer not to answer
4. **What is your religion?**
   - o None (e.g., Atheist, Agnostic, Humanist)
   - o Buddhist
   - o Christian
   - o Hindu
   - o Jewish
   - o Muslim
   - o Sikh
   - o Traditional Indigenous Spirituality
   - o Other (please specify): ____________
   - o Prefer not to answer
5. **Household composition at the time of diagnosis:**
   - o One adult without children
   - o One adult with children
   - o Two partners without children
   - o Two partners with children
   - o Other (please specify): __________________

6. **What was your children age at the time of diagnosis?**
    - o ________________

7. **At the time of diagnosis, did you receive sufficient support from your relatives and friends to cope with the stressors you experienced?**
    - o More support than expected
    - o As much support as expected
    - o Less support than expected
8. **What was your primary occupation at the time of diagnosis?**
    - o Full-time worker
    - o Part-time worker
    - o Student
    - o Retired
    - o Unemployed
    - o Looking for employment
    - o On temporary leave (e.g., medical leave)
    - o Employment insurance recipient
    - o Social assistance recipient
    - o Other (please specify):
9. **What is your highest level of education completed?**
    - o Primary
    - o Secondary
    - o College (Pre-university programs, CEGEP)
    - o Vocational training
    - o University diploma (Bachelor's or First professional degree)
    - o Master's degree
    - o Doctorate or post-doctorate
10. **What was your household income before deductions and taxes at the time of diagnosis?**
    - o Prefer not to answer
    - o Under $10,000
    - o $10,000 to $29,999
    - o $30,000 to $49,999
    - o $50,000 to $69,999
    - o $70,000 to $89,999
    - o $90,000 to $109,000
    - o $110,000 to $129,999
    - o $130,000 to $149,999
    - o $150,000 to $169,999
    - o $170,000 to $189,999
    - o $190,000 to $199,999
    - o $200,000 and over

11. Do you have a private insurance plan, other than public insurance, that reimburses the following either partially or fully?

	Yes	No	Don't know
Medications			
Imaging (e.g., X-rays, MRI, CT scans)			
Private healthcare			

12. Since your surgery, have you used your private insurance plan for the following?

	Yes	No	Don't know
Medications			
Imaging (e.g., X-rays, MRI, CT scans)			
Private healthcare			

13. At the time of diagnosis, how many days per week, on average, did you engage in physical activity for a total of 30 minutes or more, at an intensity sufficient to increase your respiratory rate?
(Examples: sports, exercise, brisk walking, cycling. Are excluded household chores or physical activity that is part of your job.)
- 0
- 1
- 2
- 3
- 4
- 5
- 6
- 7

SECTION 2: CLINICAL PROFILE

The following questions will help us learn more about the type of breast cancer you were diagnosed with, as well as the treatments you received over the past five years. Please answer while considering all the treatments you have received.

To help us better understand the timeline of events surrounding your breast cancer diagnosis, please complete the following information as accurately as possible:

1. **Dates**:
   - Date of your breast cancer diagnosis: __________________
   - Date of surgery (if you had more than one surgery, please specify the date for each): ____________________
2. **Location**:
   - Place of surgery (if you had more than one surgery, please specify the location for each): ______________________

3. **What type of breast cancer were you diagnosed with?**
    - o Early breast cancer (affecting only the breast)
    - o Locally advanced breast cancer (affecting the breast and lymph nodes)
    - o Metastatic breast cancer (affecting other organs)
    - o I don't know
4. **Are you a carrier of a genetic mutation related to your breast cancer? (e.g., BRCA1, BRCA2)**
    - o Yes
    - o No
    - o I don't know

SURGERY

5. **What type(s) of surgery have you undergone?**
   For each type of surgery listed, please select all applicable options and indicate whether the procedure was performed on the left or right side.

	Left	Right	Not applicable	I don't know
Mastectomy (complete breast removal)				
Partial mastectomy (tumor removal only)				
Sentinel lymph node removal				
Axillary lymph node removal				
Immediate breast reconstruction surgery (performed at the same time as the mastectomy)				
Delayed breast reconstruction surgery (not performed at the same time as the mastectomy)				

ADDITIONAL TREATMENTS

To treat your breast cancer, did you receive any additional treatments?
Please select all options that apply to your situation.

1. **Specify the additional treatments received, if any**:
    - o Systemic treatments (chemotherapy, immunotherapy, or targeted therapy)
    - o Local radiotherapy (breast only)
    - o Regional radiotherapy (breast and lymph nodes)
    - o Hormonal therapy (endocrine therapy, hormone therapy)
    - o I did not receive additional treatment
    - o I don't know
2. **When did you receive these additional treatments?**
   Select the option that best describes your situation:
    - o I received additional treatments **before and after** my surgery
    - o I received additional treatments **only after** my surgery
    - o I don't know

PHYSICAL SIDE EFFECTS

Among the following physical side effects, please indicate for each one how much you were bothered by it since your diagnosis.
If you have not experienced this effect, please select "Not at all."

	Not at all	A little	Moderately	A lot	Extremely
Breast pain					
Shoulder pain					

Axillary pain					
Upper limb pain (arm, forearm, hand)					
Shoulder stiffness					
Underarm stiffness					
Lymphedema (swelling in the upper limb or chest)					
Decreased shoulder range of motion					
Decreased sensitivity or paresthesia (numbness, tingling, prickling) in the chest					
Decreased sensitivity or paresthesia in the upper limb (arm, forearm, hand)					
Muscle weakness (loss of strength)					
Muscle atrophy (loss of muscle mass)					
Fatigue					
Sleep disturbances					
Shortness of breath					
Nausea					

1. **Have you been bothered by any other physical side effects not listed above?**
   If yes, please specify them in the space provided.

PSYCHOSOCIAL SIDE EFFECTS

Among the following psychosocial side effects, please indicate for each one how much you were bothered by it since your diagnosis.
If you have not experienced this side effect, please select "Not at all."

	Not at all	A little	Moderately	A lot	Extremely
A decrease in self-esteem					
Difficulties accepting body image					
Stress or anxiety					
Feeling depressed					
Difficulties relating to others					
Feeling isolated					
Feeling misunderstood					
Fear of moving					
Fear of hurting myself or hindering my recovery					
A decrease in motivation					
Memory problems					
Concentration problems					
Feeling overwhelmed					
Feeling helpless					
Feeling like a burden for others					

1. **Have you been bothered by other psychosocial side effects not listed above?**
   If yes, please specify them in the space provided.

SECTION 3: REHABILITATION NEEDS AND EXPECTATIONS

The following questions will help us better understand your physical and psychosocial rehabilitation needs throughout your care journey.

Rehabilitation care: Rehabilitation refers to a set of interventions that help people improve their ability to function in daily life and reduce the difficulties caused by a health condition, in interaction with their environment.

1. **At what stage(s) in your care journey did you have rehabilitation needs?**
   Select all that apply to your situation:
   - o Between the diagnosis and the day of my surgery
   - o Immediately after my surgery
   - o A few weeks after my surgery or during additional treatments
   - o Immediately after the end of my additional treatments
   - o A few weeks to a few months after the end of my additional treatments
   - o I had no rehabilitation needs before or after my treatments.

PRE-SURGERY NEEDS

Among the following list of needs, please indicate, on a scale from "Not at all" to "Extremely," the extent to which you felt the need for rehabilitation **before your surgery**.
If you did not have this need, please select "Not at all."

	Not at all	A little	Moderately	A lot	Extremely
Need to be informed and reassured about available rehabilitation services					
Need to be supported or guided through the rehabilitation process					
Need to discuss or meet with women who share a similar experience					
Need to access resources that will help me recover more quickly from surgery					
Need to prevent a decline in my functional abilities					
Need to improve my physical condition					

SATISFACTION WITH PRE-SURGERY NEEDS

For the rehabilitation needs selected in the previous section, please indicate to what extent these needs were met.

	Not at all	Partially	Completely
Need to be informed and reassured about available rehabilitation services			
Need to be supported or guided through the rehabilitation process			
Need to discuss or meet with women who share a similar experience			
Need to access resources that will help me recover more quickly from surgery			
Need to prevent a decline in my functional abilities			
Need to improve my physical condition			

NEEDS DURING OR AFTER TREATMENTS
Among the following list of needs, please indicate, on a scale from "Not at all" to "Extremely," the extent to which you felt the need for rehabilitation **during or after your treatments**.
If you did not have this need, please select "Not at all."

	Not at all	A little	Moderately	A lot	Extremely
Need to be informed and reassured about available rehabilitation services					
Need to be supported or guided through the rehabilitation process					
Need to discuss or meet with women who share a similar experience					
Need to access resources that will help me recover more quickly from surgery					
Need to prevent a decline in my functional abilities					
Need to improve my physical condition					

SATISFACTION WITH NEEDS DURING OR AFTER TREATMENTS
For the rehabilitation needs selected in the previous section, please indicate to what extent these needs were met.

	Not at all	Partially	Completely
Need to be informed and reassured about available rehabilitation services			
Need to be supported or guided through the rehabilitation process			
Need to discuss or meet with women who share a similar experience			
Need to access resources that will help me recover more quickly from surgery			
Need to prevent a decline in my functional abilities			
Need to improve my physical condition			

SECTION 4: EXPERIENCES IN REHABILITATION CARE

The following questions will help us better understand the nature of the rehabilitation care and services you received or would have liked to receive during your care journey.

1. **Did you receive any rehabilitation care or services during your care journey?**
   - o Yes
   - o No
   - o I don't know
2. **Please select all the rehabilitation care and services you received during your care journey, specifying when you had access to each of the listed services**.

	Between diagnosis and the day of surgery	Immediately after surgery	A few weeks after surgery or during additional treatments	After the end of additional treatments	Not applicable

Physiotherapy					
Massage therapy					
Chiropractic care					
Kinesiology					
Acupuncture					
Decongestive therapy or lymphatic drainage					
Personalized yoga or Pilates sessions					
Group yoga or Pilates sessions					
Personalized psychological care, psychotherapy, or psychosocial support					
Group psychological care, psychotherapy, or psychosocial support					
Informational workshops on self-management strategies (e.g., skincare, breast hygiene, cosmetic hygiene, infection prevention)					

3. **For each rehabilitation service selected in the previous question, please indicate your level of satisfaction** with each, based on the following criteria:

	Not at all satisfied	Unsatisfied	Neither satisfied nor unsatisfied	Satisfied	Very satisfied
Quality of care or services received					
Ease of access to care or services (availability, proximity, etc.)					
Wait time to receive care or services					
Costs of care or services					
Achievement of treatment goals					

BARRIERS TO REHABILITATION CARE

The following questions aim to identify the barriers you may have faced when accessing rehabilitation care and services.

1. **What obstacles did you encounter when accessing the rehabilitation care and services listed earlier?**
   Select all that apply to you:
   - o Lack of motivation
   - o The care or services did not meet my needs
   - o Time constraints
   - o Financial constraints
   - o Transportation constraints
   - o I didn’t have the physical capacity (e.g., sleep problems, pain, fatigue)
   - o I didn’t have the psychological capacity (e.g., concentration issues, mental overload)
   - o The care or services provided little or no benefit to my condition
   - o Other (please specify):
   - o I experienced no obstacles in accessing rehabilitation care or services

2. **Among the following rehabilitation care and services please the ones you did not use but would have liked to access. If you selected the “other” option, please specify nature of the service you would have liked to receive.**
   - o Physiotherapy
   - o Massage therapy
   - o Chiropractic
   - o Kinesiology
   - o Acupuncture
   - o Lymphatic drainage
   - o Yoga or Pilates (individualized sessions)
   - o Yoga or Pilates (group sessions)
   - o Personalized psychological care, psychotherapy, or psychosocial support
   - o Group psychological care, psychotherapy, or psychosocial support
   - o Informative workshops on self-management strategies (e.g., skincare, breast hygiene, cosmetic hygiene, infection prevention)
   - o Other
   - o I had access to all the services I needed
   - o I did not need any rehabilitation services throughout my care journey
3. **For the rehabilitation services you did not receive but would have liked to, please specify the reasons why**:
   - o The service was not available in my region
   - o The service did not meet my needs
   - o I was unaware of the availability of the service
   - o My care team did not prescribe or recommend this service
   - o Time constraints
   - o Financial constraints
   - o Transportation constraints
   - o I did not have the physical capacity (e.g., sleep problems, pain, fatigue)

- I did not have the psychological capacity (e.g., concentration issues, mental overload)
- Administrative processes to access the service were too complex
- The wait time to access the service was too long
- The time between diagnosis and surgery was too short
- I had a negative past experience with this type of service
- I wanted to prioritize my treatments
- Other (please specify):
- I prefer not to answer

INTEREST IN PHASE 2

This research project will have a second phase where we aim to further explore the results obtained from this questionnaire through group interviews.

1. **Would you like to be contacted for more information about Phase 2 of this research project?**
    - Yes
    - No
