# Supplementary material for: A Provincial Survey on the Perioperative Rehabilitation Needs and Experiences of Women Diagnosed with Breast Cancer
Source: Healthcare (Basel). 2025 Dec 10;13(24):3239. doi: 10.3390/healthcare13243239 (PMC12733279; doi:10.3390/healthcare13243239)
Supplement: Supplementary file 1 [file healthcare-13-03239-s001.zip › SupplementaryFile S3.pdf]

Additional File II – Barriers to rehabilitation

Patients who did use rehabilitation services		
Barriers faced **when using rehabilitation services** (N=245)		
None	70 (28.6)	-
Financial constraints	67 (27.3)	
Impaired physical function	64 (26.1)	
Loss of motivation	54 (22.0)	
Other	41 (16.7)	
Impaired psychological function	32 (13.1)	
The service did not meet my expectations	29 (11.8)	
Time constraints	28 (11.4)	
The service was of little benefit to my condition	24 (9.8)	
Transportation issues	23 (9.4)	
Services patients would have liked to have access to (N= 245)		
Massage therapy	55 (22.4)	
Informative workshops	53 (21.6)	
Yoga - solo	49 (20.0)	
Acupuncture	48 (19.6)	
Kinesiology	47 (19.2)	
Psychotherapy – group sessions	43 (17.6)	
Lymphatic drainage	42 (17.1)	
Psychotherapy - solo	41 (16.7)	
Physiotherapy	37 (15.1)	
Yoga – group sessions	29 (11.8)	
Other	25 (10.2)	
Chiropractic	17 (6.9)	
Had access to all the services they needed	9 (3.7)	
Barriers to the use of desired rehabilitation services (N=231)		
Has not been prescribed or recommended	77 (32.6)	-
I was not aware that this service existed	68 (28.8)	
Financial constraints	49 (20.8)	
The service was not available in my region	38 (16.1)	
Waiting lists	22 (9.3)	
Impaired physical function	15 (6.4)	
The service did not meet my expectations	14 (5.9)	
Wanted to focus on my treatments	13 (5.5)	
Other	13 (5.5)	
Impaired psychological function	9 (3.8)	

<table>
<tr><td>Time constraints</td><td>8 (3.4)</td><td rowspan="6"></td></tr>
<tr><td>Complex administrative procedures</td><td>8 (3.4)</td></tr>
<tr><td>Prefer not to answer</td><td>7 (3.0)</td></tr>
<tr><td>Transportation issues</td><td>6 (2.5)</td></tr>
<tr><td>Short delay from diagnosis to surgery</td><td>2 (0.8)</td></tr>
<tr><td>Negative past experiences</td><td>2 (0.8)</td></tr>
<tr><td colspan="3">Patients who did not use rehabilitation services</td></tr>
<tr><td colspan="3">Services they would have liked to have access to (N= 196)</td></tr>
<tr><td>Massage therapy</td><td>83 (42.3)</td><td rowspan="13">4 (2.0)</td></tr>
<tr><td>Physiotherapy</td><td>66 (33.7)</td></tr>
<tr><td>Psychotherapy - solo</td><td>65 (33.2)</td></tr>
<tr><td>Informative workshops</td><td>62 (31.6)</td></tr>
<tr><td>Had access to all the services they needed</td><td>48 (24.5)</td></tr>
<tr><td>Lymphatic drainage</td><td>47 (24.0)</td></tr>
<tr><td>Yoga – group sessions</td><td>45 (23.0)</td></tr>
<tr><td>Kinesiology</td><td>44 (22.4)</td></tr>
<tr><td>Yoga - solo</td><td>42 (21.4)</td></tr>
<tr><td>Acupuncture</td><td>39 (19.9)</td></tr>
<tr><td>Psychotherapy – group sessions</td><td>39 (19.9)</td></tr>
<tr><td>Chiropractic</td><td>27 (13.8)</td></tr>
<tr><td>Other</td><td>17 (8.7)</td></tr>
<tr><td colspan="3">Patients who did not use rehabilitation services - Barriers to access to rehabilitation services (N=144)</td></tr>
<tr><td>I was not aware that this service existed</td><td>60 (41.7)</td><td></td></tr>
<tr><td>Has not been prescribed or recommended</td><td>55 (38.2)</td><td></td></tr>
<tr><td>Financial constraints</td><td>24 (16.7)</td><td></td></tr>
<tr><td>The service was not available in my region</td><td>16 (11.1)</td><td rowspan="11">1 (0.6)</td></tr>
<tr><td>Impaired physical function</td><td>14 (9.7)</td></tr>
<tr><td>Waiting lists</td><td>12 (8.3)</td></tr>
<tr><td>Other</td><td>12 (8.3)</td></tr>
<tr><td>Impaired psychological function</td><td>8 (5.6)</td></tr>
<tr><td>Wanted to focus on my treatments</td><td>7 (4.9)</td></tr>
<tr><td>Complex administrative procedures</td><td>6 (4.2)</td></tr>
<tr><td>Prefer not to answer</td><td>5 (3.5)</td></tr>
<tr><td>Short delay from diagnosis to surgery</td><td>4 (2.7)</td></tr>
<tr><td>The service did not meet my expectations</td><td>3 (2.1)</td></tr>
</table>

Additional File II – Barriers to rehabilitation

Time constraints	3 (2.1)	
Transportation issues	3 (2.1)	
Negative past experiences	2 (1.4)	
