# Supplementary material for: A Provincial Survey on the Perioperative Rehabilitation Needs and Experiences of Women Diagnosed with Breast Cancer
Source: Healthcare (Basel). 2025 Dec 10;13(24):3239. doi: 10.3390/healthcare13243239 (PMC12733279; doi:10.3390/healthcare13243239)
Supplement: Supplementary file 1 [file healthcare-13-03239-s001.zip › SupplementaryFile S2.pdf]

Additional File I – Rehabilitation needs

Timing of rehabilitation needs (N=519)	N (%)	Missing values N (%)
Rehabilitation needs (timing)		
From diagnosis to surgery	80 (15.4)	63 (12.1)
Immediately after surgery	135 (26.0)	
A few weeks after surgery or during complementary treatments	218 (42.0)	
Immediately after the end of complementary treatments	98 (18.9)	
A few weeks to a few months after the end of complementary treatments	111 (21.4)	
No rehabilitation needs	112 (21.6)	
Rehabilitation needs **before surgery** (N=80)		
Being informed and reassured about available services		1 (1.3)
Not at all	1 (1.3)	
Slightly	10 (8.0)	
Moderately	17 (21.3)	
Very	30 (37.5)	
Extremely	20 (25.0)	
Be guided and supported in the rehabilitation process		1 (1.3)
Not at all	6 (7.5)	
Slightly	14 (17.5)	
Moderately	21 (26.3)	
Very	22 (27.5)	
Extremely	16 (20.0)	
To discuss or meet women who face a similar reality to mine		1 (1.3)
Not at all	16 (20.0)	
Slightly	14 (17.5)	
Moderately	15 (18.8)	
Very	20 (25.0)	
Extremely	14 (17.5)	
Obtaining resources to help me recover more quickly from surgery		1 (1.3)
Not at all	5 (6.3)	
Slightly	11 (13.8)	
Moderately	10 (8.0)	
Very	31 (38.8)	
Extremely	22 (27.5)	
Preventing loss of functional capacities		1 (1.3)
Not at all	5 (6.3)	
Slightly	11 (13.8)	
Moderately	13 (16.3)	
Very	27 (33.8)	
Extremely	23 (28.8)	

Additional File I – Rehabilitation needs

Improving physical condition		1 (1.3)
Not at all	5 (6.3)	
Slightly	14 (17.5)	
Moderately	18 (22.5)	
Very	27 (33.8)	
Extremely	15 (18.8)	

Rehabilitation needs **during or after treatments** (N= 396)		
Being informed and reassured about available services		68 (17.2)
Not at all	32 (8.1)	
Slightly	74 (18.7)	
Moderately	75 (18.9)	
Very	101 (25.5)	
Extremely	46 (11.6)	
Be guided and supported in the rehabilitation process		68 (17.2)
Not at all	54 (13.6)	
Slightly	77 (19.4)	
Moderately	70 (17.7)	
Very	90 (22.7)	
Extremely	37 (9.3)	
To discuss or meet women who face a similar reality to mine		68 (17.2)
Not at all	66 (16.7)	
Slightly	75 (18.9)	
Moderately	73 (18.4)	
Very	63 (15.9)	
Extremely	51 (12.9)	
Obtaining resources to help me recover more quickly from surgery		68 (17.2)
Not at all	41 (10.4)	
Slightly	64 (16.2)	
Moderately	71 (17.9)	
Very	98 (24.7)	
Extremely	54 (13.6)	
Preventing loss of functional capacities		68 (17.2)
Not at all	47 (11.9)	
Slightly	55 (13.9)	
Moderately	62 (15.7)	
Very	102 (25.8)	
Extremely	62 (15.7)	
Improving physical condition		68 (17.2)
Not at all	24 (6.1)	
Slightly	43 (10.9)	
Moderately	76 (19.2)	
Very	120 (30.3)	
Extremely	65 (16.4)	
